# Anti-Inflammatory Effects of Ang-(1-7) in Ameliorating HFD-Induced Renal Injury through LDLr-SREBP2-SCAP Pathway

**DOI:** 10.1371/journal.pone.0136187

**Published:** 2015-08-20

**Authors:** Yaning Zheng, Lin Tang, Wenhan Huang, Ruyu Yan, Feifeng Ren, Lei Luo, Ling Zhang

**Affiliations:** Department of Nephrology, Second Affiliated Hospital of Chongqing Medical University, Chongqing, 400010, China; The University of Manchester, UNITED KINGDOM

## Abstract

The angiotensin converting enzyme 2-angiotensin-(1–7)-Mas axis (ACE2-Ang-(1–7)-Mas axis) is reported to participate in lipid metabolism in kidney, but its precise effects and underlying mechanisms remain unknown. We hypothesized that Ang-(1–7) reduces lipid accumulation and improves renal injury through the low density lipoprotein receptor–sterol regulatory element binding proteins 2–SREBP cleavage activating protein (LDLr-SREBP2-SCAP) system by suppressing inflammation in high fat diet (HFD)-fed mice. In this study, male C57BL/6 mice were randomized into four groups: STD (standard diet)+saline, HFD+saline, HFD+Ang-(1–7) and STD+Ang-(1–7). After 10 weeks of feeding, mice were administered Ang-(1–7) or saline for two weeks. We found that high inflammation status induced by HFD disrupted the LDLr-SREBP2-SCAP feedback system. Treatment of mice fed a high-fat diet with Ang-(1–7) induced significant improvement in inflammatory status, following the downregulation of LDLr, SREBP2 and SCAP, and then, decreased lipid deposition in kidney and improved renal injury. In conclusion, the anti-inflammatory effect of Ang-(1–7) alleviates renal injury triggered by lipid metabolic disorders through a LDLr- SREBP2-SCAP pathway.

## Introduction

The prevalence of obesity is dramatically increasing worldwide. According to World Health Organization statistics, more than 1.4 billion adults over 20 are overweight and about of 154 million adults are obese (body mass index >30 kg/m^2^) [1]. Obesity is considered the major risk factor for dyslipidemia, atherosclerosis, hypertension and insulin resistance, which lead to the development of cardiocerebrovascular disease and diabetes. Additionally, dyslipidemia induced by obesity is actively involved in the onset and exacerbation of kidney disease [2–5]. Various obesity models, including lipid disruption, have been developed to demonstrate renal injury, such as glomerular hypertrophy, thickening of glomerular basement membrane, mesangial matrix expansion and increased renal inflammation, which contribute to albuminuria, renal function impairment, and eventually, glomerulosclerosis and tubulointerstitial fibrosis [2,3,6–8]. However, the signaling pathways resulting in lipid accumulation and damage in kidney are not well understood at present.

Low density lipoprotein receptor (LDLr), the primary receptor in maintenance of the balance of lipid metabolism, is highly expressed in kidney [9,10] LDLr, sterol-regulatory element-binding protein-2 (SREBP2) and SREBP-cleavage activating protein (SCAP) form a tight feedback loop which forms the pivotal system in regulating absorption of LDL cholesterol. When cells lack or contain excess cholesterol relative to that required for their physological needs, expression of the LDLr gene is up- or downregulated, which is mediated by translocation of SREBP2. This protein is transported from endoplasmic reticulum (ER) to the Golgi for cleavage, following which the N-terminal region enters the nucleus and binds to the sterol regulatory element (SRE-1) site within the LDLr promoter, activating gene transcription by SCAP. Conversely, LDLr gene transcription is suppressed when the SREBP2-SCAP complex remains in the ER in a high-cholesterol environment [11]. However, inflammation disrupts this tight LDLr-SREBP2-SCAP negative feedback system, subsequently exacerbating lipid accumulation in kidney [9,10,12].

Inflammation is correlated with the progression of various diseases, including chronic kidney diseases, diabetes and cardiovascular diseases, [13–15] and furthermore, plays a critical role in lipid metabolic disorders. In terms of the association between inflammation and lipid metabolism, on the one hand, dyslipidemia induces circulating inflammation and partial inflammation [2,16,17], while conversely, inflammation stress exacerbates lipid uptake [9,12]. Increasing research has thus focused on the potential beneficial effects of anti-inflammation therapy on dyslipidemia [16,18].

The ACE2-Ang-(1–7)-Mas axis, a recently identified branch of the renin-angiotensin system (RAS), has been investigated in several studies [19–21]. Ang-(1–7), a major component in this axis, is formed primarily from ACE2 metabolizing Ang-II mainly present in the cardiovascular system and kidney. In kidney, Ang-(1–7) is predominantly formed in the renal cortex [22]. The concentrations of Ang-(1–7) and AngII are comparable in kidney, and Ang-(1–7) is detectable in human urine [23]. Ang-(1–7) activity on the specific G protein-coupled Mas receptor induces vasorelaxation, antihypertension, antithrombosis, antifibrosis and anti-inflammation, opposite to the effects of the ACE/AngII/AT1 axis [24–27]. In addition, the ACE2-Ang-(1–7)-Mas axis induces significant amelioration of metabolic syndrome [28,29]. Substantial evidence supports the theory that the ACE2-Ang-(1–7)-Mas axis plays critical roles in renal function, inducing reduction of proteinuria, improvement of creatinine clearance and alleviation of renal structure [30–32]. Zhang *et al*. [33] further demonstrated that Ang-(1–7) attenuates progression of renal injury in streptozotocin-induced diabetic rats to a higher extent than angiotensin receptor blockade, despite similar blood glucose levels. In view of these findings, we hypothesized that exogenous Ang-(1–7) can effectively ameliorate lipid-induced renal damage. Previously, our group demonstrated for the first time that Ang-(1–7) alleviates absorption of lipid in human mesangial cells (HMCs) through regulating the LDL-SREBP2-SCAP feedback system [34]. However, the precise effects and mechanisms of action of Ang-(1–7) on renal damage induced by lipid *in vivo* are yet to be established.

The main aim of this study was to evaluate whether injury induced by disruption of lipid metabolism in kidney can be prevented by the anti-inflammatory effect of Ang-(1–7).

## Results

### Body weight and food intake changes in experimental animals

**Fig 1:**
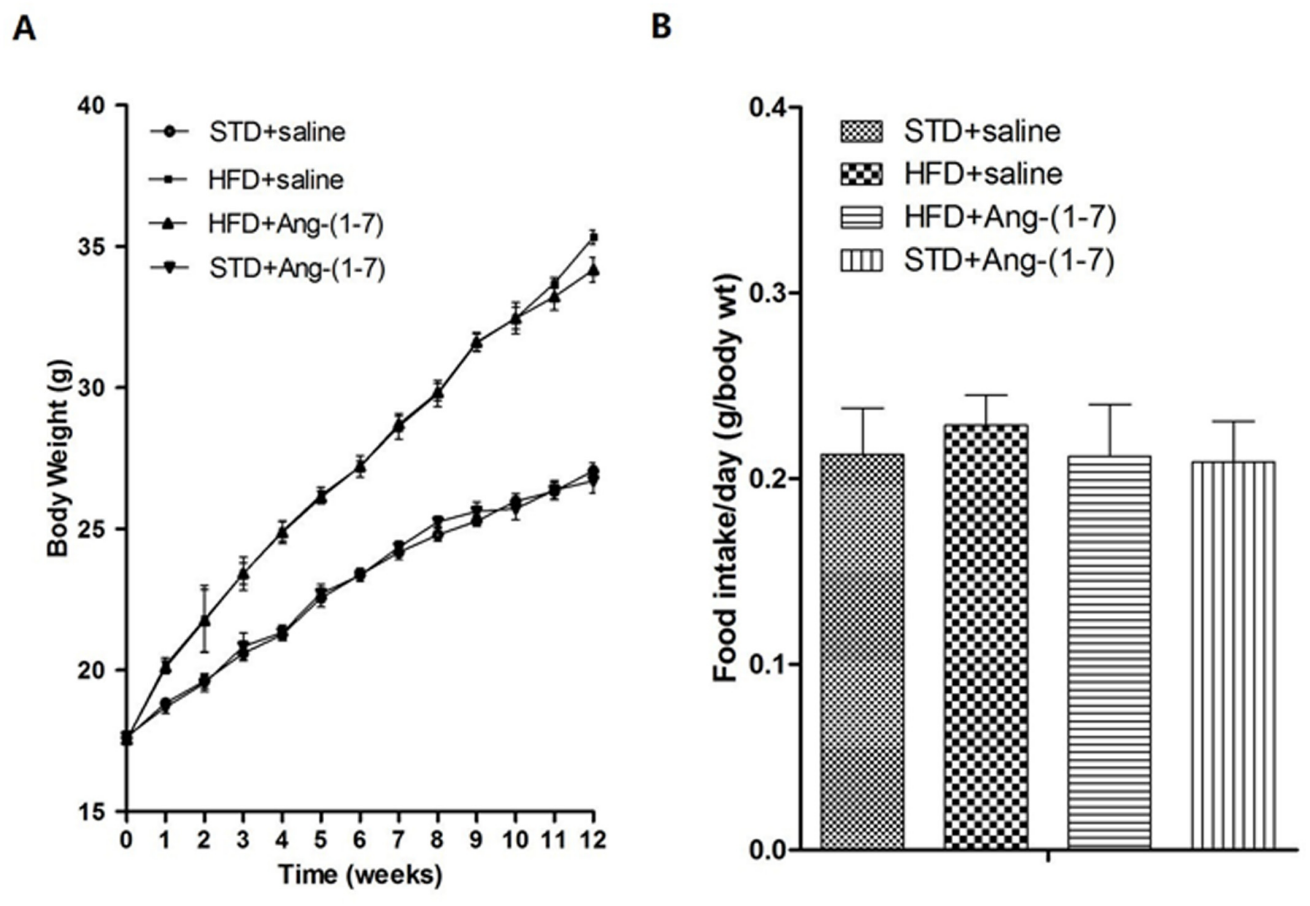
Changes in body weight and food intake. A: Average weekly body weights of male C57BL/6 mice in all groups. Values are expressed as means ± SD (n = 10). B: Food intake was measured to obtain food efficiency. Values are expressed as means ± SD (n = 12).

Mice were divided into four groups: STD+saline, HFD+ saline, HFD + Ang-(1–7) and STD + Ang-(1–7). During the study period, no mortality or morbidity was observed in any group, and all animals gained weight (Fig 1A). Differences in food intake (g/body weitht) was not observed between groups (Fig 1B). Before implantation of osmotic pumps, significant body gain was recorded in high fat diet-fed mice. At the end of the experimental period, body weights of mice in HFD+Ang-(1–7) were significantly reduced (HFD+saline: 35.33 ± 0.25 g, *P*<0.05, vs. HFD+Ang-(1–7): 34.18 ± 0.44 g, n = 10), while no differences were observed between STD+saline and STD+Ang-(1–7) (STD+saline: 27.06 ± 0.27 g vs. STD+Ang-(1–7): 26.7 ± 0.43 g, n = 10).

### Ang-(1–7) ameliorates dyslipidemia induced by HFD

Changes in lipid levels in each group are presented in Table 1 (n = 10). We observed a significant increase in TC, TG and LDL in HFD mice. Treatment of HFD mice with Ang-(1–7) led to a marked decrease in plasma levels of TC, TG and LDL.

**Table 1:** Serum and urine biochemical parameters.

Item	STD+saline	HFD+saline	HFD+Ang-(1–7)	STD+Ang-(1–7)
TC (mmol/l)	2.26±0.41	4.04±0.33^a^	3.37±0.28^b^	2.29±0.31
TG (mmol/l)	0.41±0.06	0.71±0.11^a^	0.58±0.05^b^	0.42±0.05
LDL (mmol/l)	0.24±0.07	0.56±0.08^a^	0.49±0.03^b^	0.25±0.05
BUN (mmol/l)	4.04±0.53	6.96±0.91^a^	4.91±0.68^b^	4.15±0.44
Scr (umol/l)	26.37±2.31	50.91±6.55^a^	36.55±3.25^b^	26.34±2.00
Urinary albumin (mg/24h)	0.13±0.01	0.45±0.03^a^	0.26±0.02^b^	0.13±0.02
CRP (mg/l)	0.11±0.03	0.89±0.39^a^	0.52±0.11^b^	0.13±0.05
TNF-α(pg/ml)	89.36±19.21	275.93±46.33^a^	159.08±19.64^b^	94.39±18.81
IL-6 (pg/ml)	23.64±2.87	64.21±9.85^a^	42.31±6.86^b^	23.69±2.42

Serum and urine biochemical parameters in all groups. Values are expressed as means ±SD.

^a^ *P*<0.05 HFD+saline vs. STD+saline,

^b^ *P*<0.05 HFD+saline vs. HFD+Ang-(1–7).

### Ang-(1–7) reduces inflammation induced by HFD

**Fig 2:**
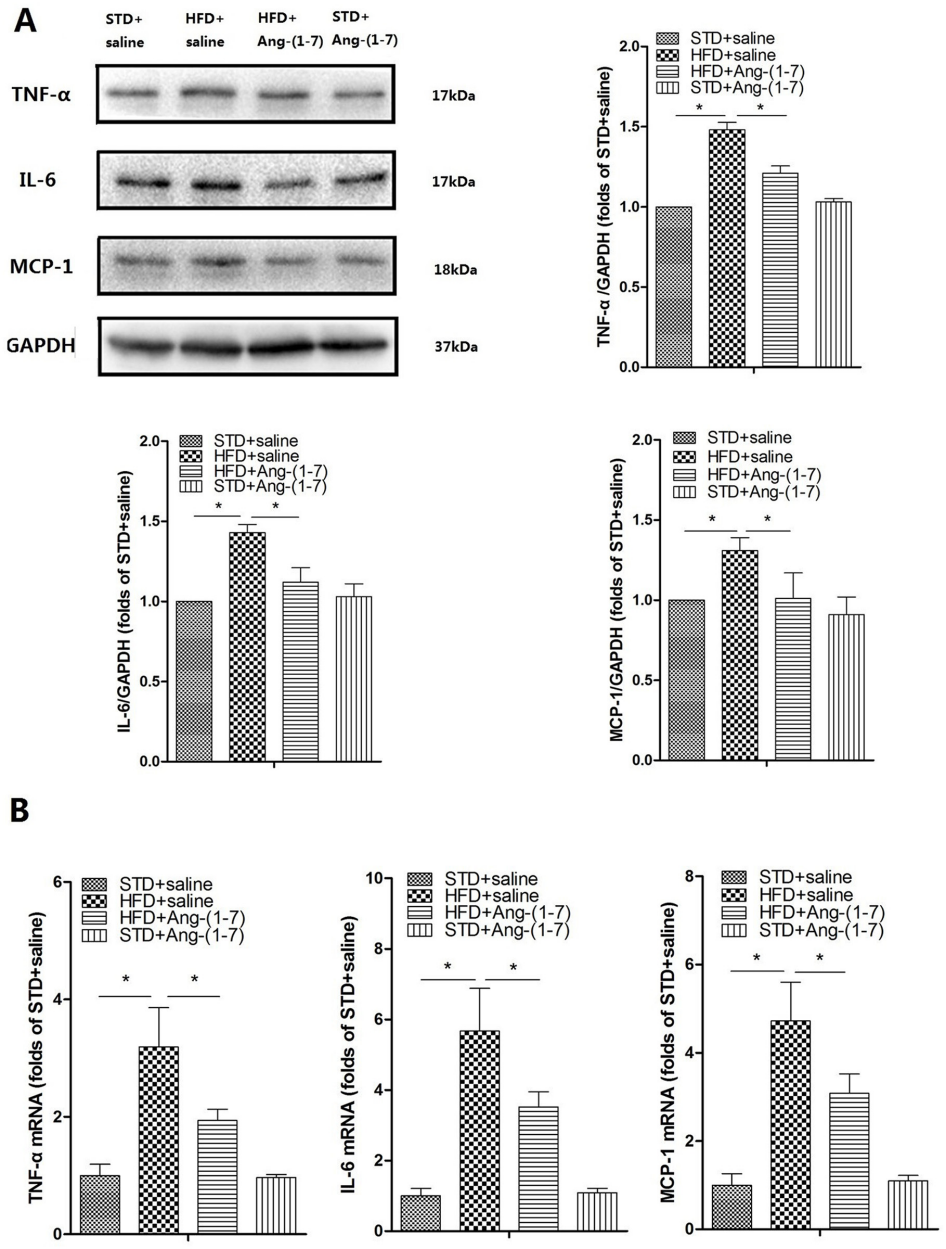
Ang-(1–7) mitigates HFD-induced high inflammation status in kidney. A: Representative immunoblots for TNF-α, IL-6 and MCP-1. Representative quantitative analysis of TNF-α, IL-6, and MCP-1. Values are expressed as means ± SD (n = 6). B: TNF-α, IL-6 and MCP-1 mRNA levels were measured using real-time PCR. The GAPDH gene served as the internal control. Values are expressed as means ± SD (n = 6), *P<0.05.

**Fig 3:**
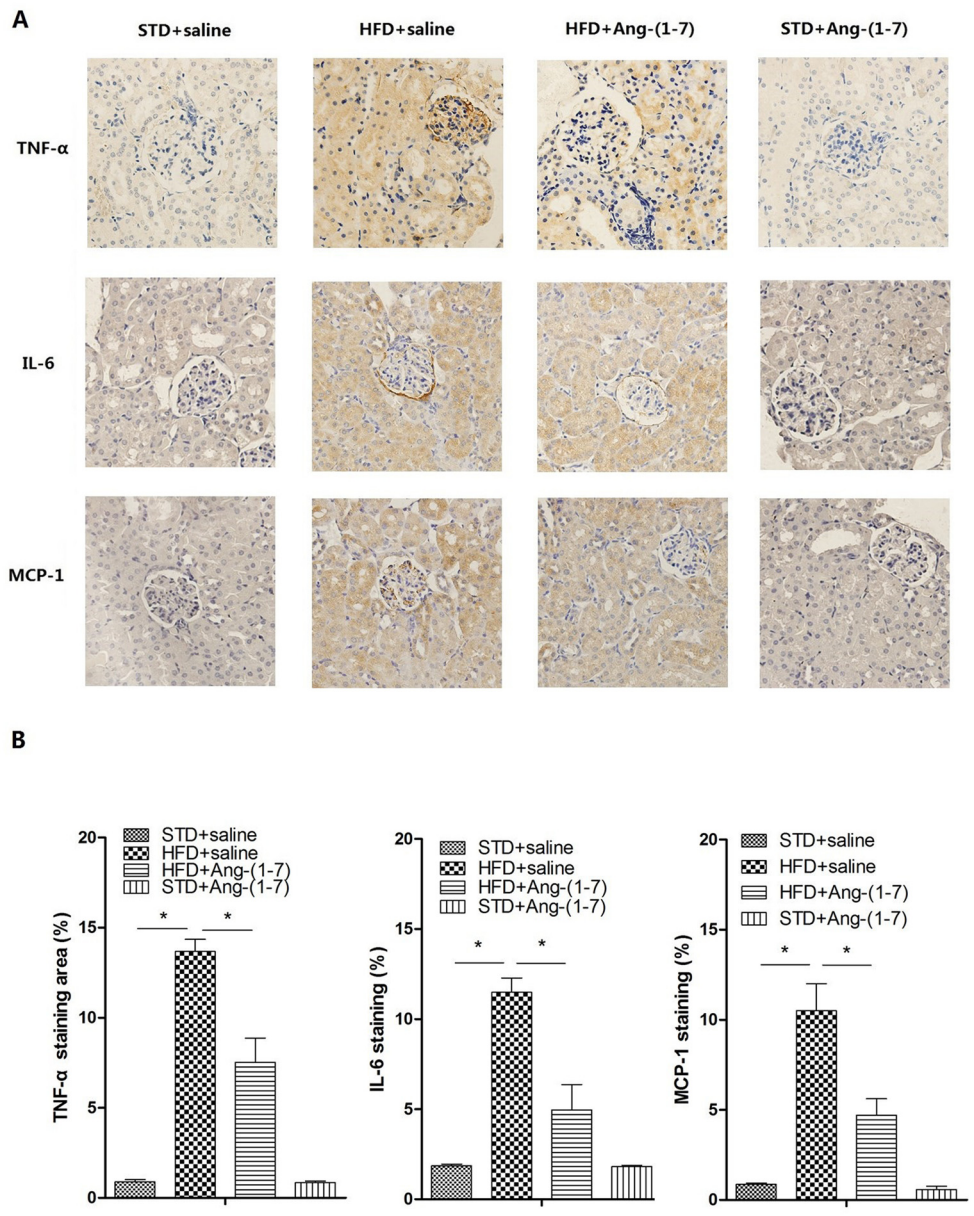
Immunohistochemical staining for inflammation in renal sections. Ang-(1–7) inhibits HFD-induced TNF-α, IL-6 and MCP-1 expression. A: Representative immunostaining of TNF-α, IL-6 and MCP-1 in all groups (magnification 400×) B: Quantification of TNF-α, IL-6 and MCP-1 staining was performed using Image-Pro Plus v 6.0. Values are expressed as means ± SD (n = 6), *P<0.05.

At the end of the experimental period, HFD+saline showed a significant increase in CRP, TNF-α, and IL-6 levels in serum (Table 1, n = 10). Immunohistochemistry, Western blot and real-time PCR analyses collectively demonstrated that compared with STD+saline, inflammatory markers (TNF-α, IL-6, MCP-1) in kidney were significantly increased in HFD+saline, indicating that hyper inflammatory status is induced by HFD *in vivo* (Figs 2 and 3 respectively, n = 6). Treatment of HFD-fed mice with Ang-(1–7) significantly reduced inflammation in the systemic circulation and renal tissue. HFD+Ang-(1–7) showed significant reduction of serum CRP, TNF-α, and IL-6 (Table 1). Immunohistochemistry, Western blot and real-time PCR findings revealed a marked decrease in the levels of inflammatory markers (TNF-α, IL-6, MCP-1) in renal tissue of HFD+Ang-(1–7) mice (Figs 2 and 3 respectively, n = 6).

### Ang-(1–7) alleviates lipid deposition in kidney via the LDLr-SREBP2-SCAP pathway

**Fig 4:**
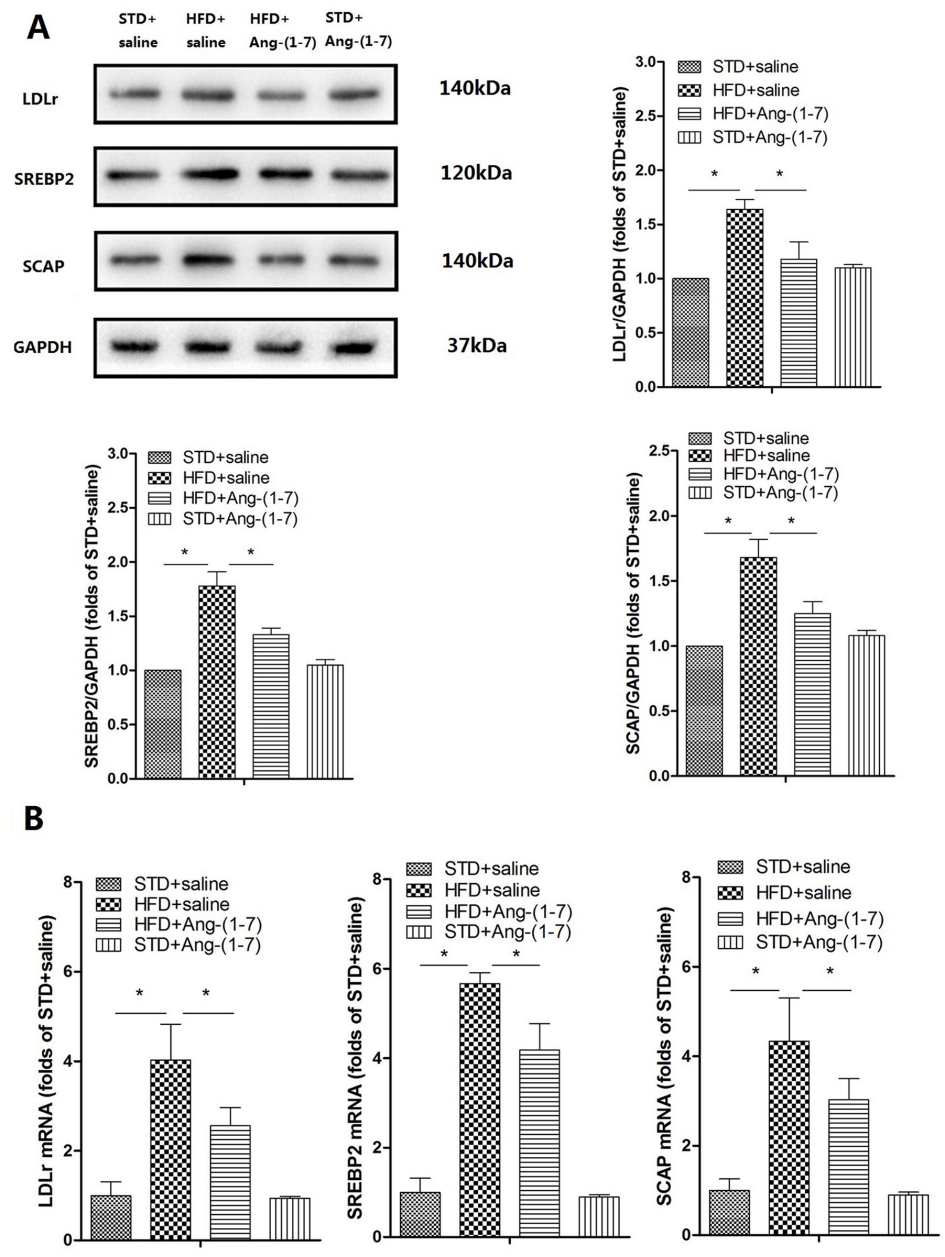
Ang-(1–7) inhibits HFD-induced LDLr, SREBP2 and SCAP expression in kidney. A: Representative immunoblots for LDLr, SREBP2 and SCAP. Representative quantitative analysis of LDLr, SREBP2 and SCAP. Values are expressed as means ± SD (n = 6). B: Real-time PCR was applied to measure mRNA levels. The GAPDH gene served as the internal control. Values are expressed as means ± SD (n = 6), *P<0.05.

**Fig 5:**
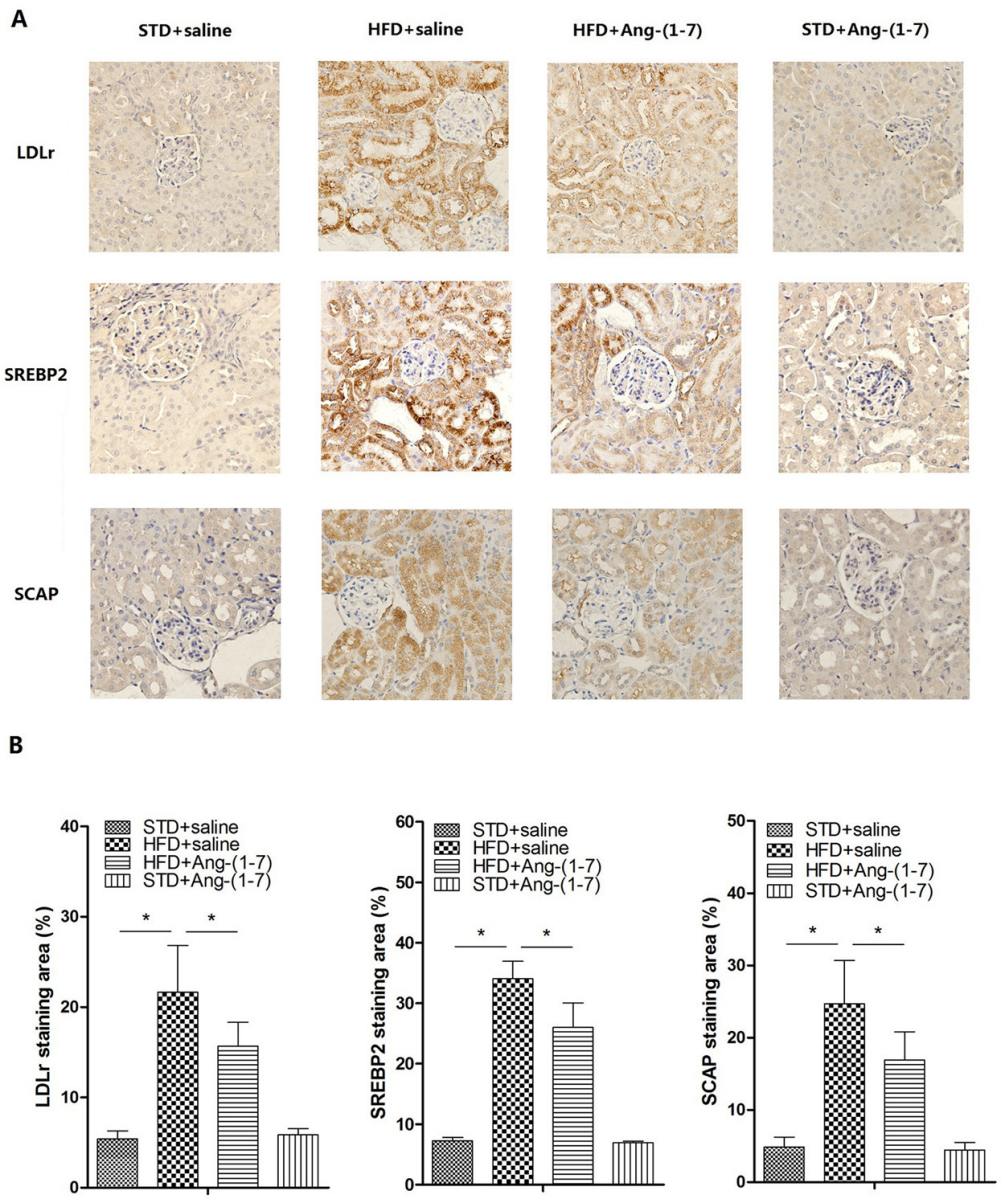
Immunohistochemical staining of lipid regulation in the renal section. Ang-(1–7) inhibits HFD-induced LDLr, SREBP2 and SCAP expression. A: Representative immunostaining of LDLr, SREBP2 and SCAP in all groups (magnification 400×) B: Quantification of LDLr, SREBP2 and SCAP staining evaluated with Image-Pro Plus v 6.0. Values are expressed as means ± SD (n = 6), *P<0.05.

**Fig 6:**
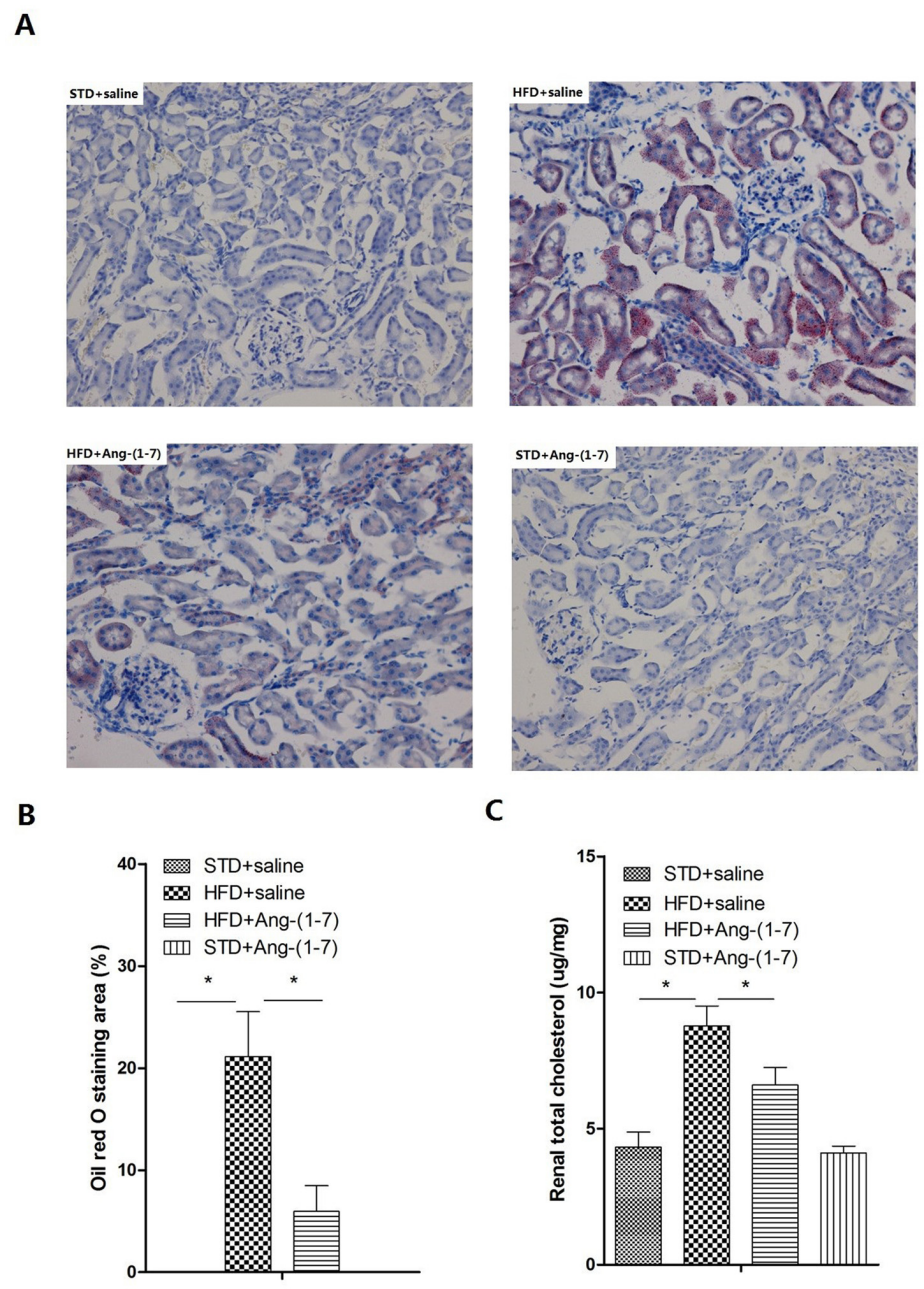
Renal lipid accumulation in mice. Ang-(1–7) ameliorates HFD-induced renal lipid deposition. A: Oil Red O staining (magnification 200×). Representative Oil Red O staining in renal sections. Red spots signify lipid drops. HFD induces significant accumulation of lipid drops in kidney, which is reduced upon treatment with Ang-(1–7). B: Representative quantitative analysis of Oil Red O staining. Values are expressed as means ± SD (n = 6). C: Measurement of renal total cholesterol as described in Materials and Methods. Values are expressed as means ± SD (n = 6), *P<0.05.

Expression patterns of LDLr, SREBP2 and SCAP in kidney were analyzed via Western blot, real-time PCR and immunohistochemistry analyses (Figs 4 and 5 respectively, n = 6). Expression of LDLr, SREBP2 and SCAP in HFD-fed mice was markedly increased, compared with that in normal mice. Notably, treatment of HFD-fed mice with Ang-(1–7) led to significant reduction of expression of these proteins. Lipid accumulation was detected with Oil red O staining and assessment of renal total cholesterol. Several lipid droplets were deposited in HFD+saline mice whereas none were found in STD+saline (Fig 6A and 6B). In addition, lower numbers of lipid droplets were observed in HFD+Ang-(1–7), relative to HFD+saline. As illustrated in Fig 6C, a significant increase in renal total cholesterol was evident in HFD+saline, compared with STD+saline (STD+saline: 4.32 ± 0.55 μg/mg, *P*<0.05, vs. HFD+saline: 8.77 ± 0.74 μg/mg, n = 6). Notably, renal cholesterol was significantly reduced in HFD+Ang-(1–7), compared with HFD+saline mice (HFD+saline: 8.77 ± 0.74 μg/mg *P*<0.05 vs. HFD+Ang-(1–7): 6.61 ± 0.64, n = 6).

### Ang-(1–7) mitigates HFD-induced structural damage of glomeruli and tubules

**Fig 7:**
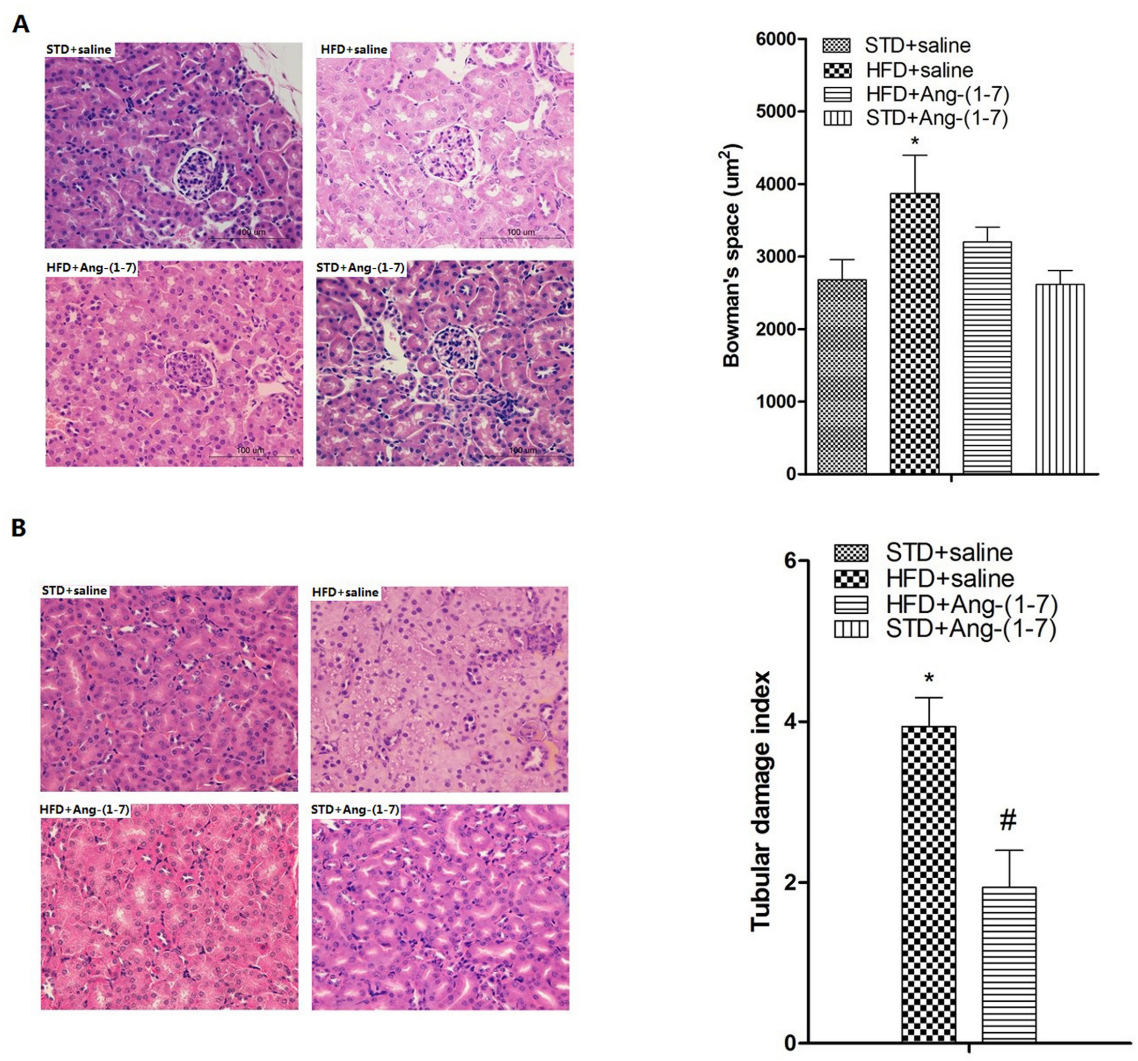
HE staining of a representative renal section. Ang-(1–7) improves HFD-induced glomerular and tubular damage. A: Images of glomerulus and representative quantitative analysis of Bowman’s space. Scale bar is 100 μm. Values are expressed as means ± SD (n = 6) B: Images of tubules and representative quantitative analysis of tubular damage index. Original magnification 200×. Values are expressed as means ± SD (n = 6), *P<0.05.

**Fig 8:**
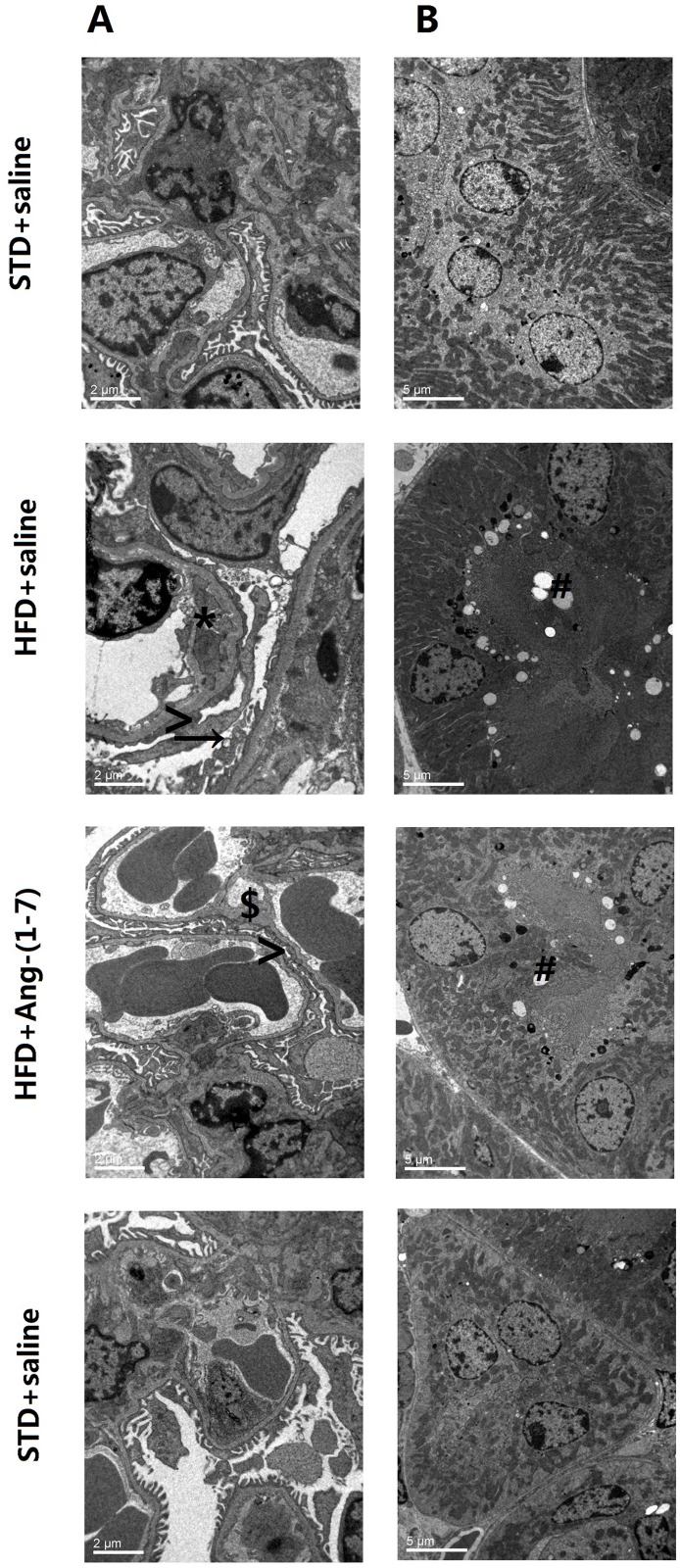
Findings of transmission electron microscopy analysis. Ang-(1–7) improves HFD-induced GBM injury and lipid droplet accumulation in tubules. Photographs of podocyte fusion (>), mesangial cell cytoplasm insert in GBM (*), podocytes absorbing lipid droplets (→), lipid droplet accumulation in tubules (#), thickening of GBM ($) from all Groups. A: Glomeruli (magnification 4000×) B: tubules (magnification 8000×).

The effects of HFD and subsequent treatment with Ang-(1–7) on renal structure were examined via histopathology. HE staining revealed that glomerular size of HFD+saline is significantly higher than that of STD+saline, with increased patches of tubular vacuolation (Fig 7, n = 6). We additionally observed severe tubular cell swelling, tubular dilatation, brush border loss and nuclear loss in HFD+saline. The damage induced by HFD in glomeruli and tubules was prevented by Ang-(1–7). TEM analysis revealed severe podocyte fusion, mesangial cell cytoplasm insert in glomerular basement membrane (GBM), podocytes filled with lipid uptake droplets, and accumulation of numerous lipid droplets in tubules in HFD+saline, but only slight podocyte fusion, minor thickening of GBM, and limited lipid droplet accumulation in tubules in HFD+Ang-(1–7) (Fig 8). The histopathologic findings clearly indicate that structural damage in kidneys of HFD-fed mice is protected by Ang-(1–7).

### Ang-(1–7) alleviates HFD-induced renal injury

HFD-induced renal injury was detected based on serum and urine biochemical parameters, including BUN, Scr and urinary albumin levels (Table 1, n = 10). Compared with STD+saline, BUN, Scr and urinary albumin were significantly increased in HFD-fed mice. Renal injury of HFD mice was prevented by administration of Ang-(1–7).

In contrast, treatment of STD-fed mice with Ang-(1–7) did not induce alterations in body weight, serum lipid level, glycemia, inflammation or renal function, compared with STD+saline.

## Discussion

HFD induces obesity directly and causes a series of diseases correlated with lipid metabolism disorder [35,36]. Obesity-related renal disease is characterized by proteinuria, hypertrophy and glomerulosclerosis [3,4,37]. Increased body mass index and high fat intake are also high-affinity indicators for inflammation, which disrupt the state of lipid metabolism and further aggravate renal damage [2,9]. Our experiments showed that a high-fat diet induces a time-dependent increase in body weight and dramatically enhances the inflammatory index in plasma and kidney, potential features of human lipid metabolic disorders. Under the high inflammatory conditions induced by dyslipidemia, the tight LDLr-SREBP2-SCAP feedback loop critical for maintaining lipid uptake and synthesis is disrupted. We previously demonstrated that a single trigger of LDL can stimulate LDLr-SREBP2-SCAP feedback in HMCs, and Ang-(1–7) exacerbates this feedback system to inhibit accumulation of LDL [34]. However, in this study, we showed that a high-fat diet in mice causes lipid deposition in kidney through upregulation of the LDLr-SREBP2-SCAP pathway, opposite to the *in vitro* findings, and Ang-(1–7) improves this regulation. Interestingly, Ang-(1–7) ameliorated lipid accumulation in kidney through reducing inflammation. To our knowledge, this is the first study to reveal that the anti-inflammation effect of Ang-(1–7) modulates renal lipid metabolism through the LDLr-SREBP2-SCAP pathway, consequently reducing renal damage induced by lipid.

Glomerular or interstitial inflammation is known to play a critical role in renal damage. MCP-1 is the only known inflammatory marker showing increased levels in early HFD-induced kidney disease, which further recruits macrophages that enhance the release of inflammatory mediators, including TNF-α and IL-6 [2]. In HFD mice, upregulation of TNF-α has been reported to lower PPAR-ð in kidney. Renal injury induced by HFD was prevented by upregulation of PPAR-ð through blockade of the TNF-α receptor [38]. Moreover, blocking the IL-6 receptor in ApoE^-/-^ mice led to notable improvement of proteinuria, renal lipid deposition and mesangial cell proliferation [39]. Thus, release of inflammatory cytokines appears to play an essential role in hyperlipidemia-induced renal injury. Numerous studies to date have shown that the ACE2-Ang-(1–7)-Mas axis exerts inhibitory effects on inflammation in various diseases [27,40,41]. We observed significant reduction of inflammatory markers in plasma, including CRP, TNF-α and IL-6, and renal inflammatory cytokines, including IL-6, TNF-α, and MCP-1 in high fat diet-fed mice administered Ang-(1–7) (144 μg/kg·day) for two weeks, supporting a strong anti-inflammatory effect of Ang-(1–7) in both circulating blood and kidney. Meanwhile, infusion of Ang-(1–7) in healthy male C57BL/6 mice did not induce renal inflammatory changes. In keeping with these results, Ang-(1–7) (144 μg/kg·day) administered for two weeks exerted a renoprotective effect in Zucker diabetic fatty (ZDF) rats following reduction in the levels of inflammation markers [31]. In an earlier investigation, intraperitoneal administration of Ang-(1–7) (600 μg/kg·day) in spontaneously hypertensive rats induced a significant decrease in renal inflammatory cytokine levels, but did not affect cytokine levels in healthy rats [32]. In contrast, Esteban *et al*. [42] demonstrated that infusion of Ang-(1–7) (15 μg/mouse per day) in models of both unilateral ureteral obstruction and ischemia/reperfusion mice aggravated the renal inflammation response. Furthermore, infusion of Ang-(1–7) (45 μg/mouse per day) alone into healthy wild-type mice induced inflammatory cell infiltration into kidney. Taken together, differences in doses, experimental methods and animal models may contribute to these discrepancies.

Here, renal injury induced by lipid deposition was characterized by a significant increase in urinary albumin, blood urea nitrogen, serum creatinine, and morphological changes in HFD mice. HE and Oil Red O staining and TEM observations disclosed lipid droplet deposition in renal sections of HFD mice. Furthermore, upregulation of LDLr, SREBP2 and SCAP in renal tissues of HFD mice induced cholesterol uptake, which possibly contributed to renal cholesterol accumulation. We previously reported opposite regulation of the LDLr-SREBP2-SCAP pathway stimulated by LDL *in vitro*. However, we observed a significant increase in LDLr, SREBP2 and SCAP in HFD mice in the current study. One possibility is that systemic inflammation induced by HFD disrupts the LDLr-SREBP2-SCAP pathway, leading to exacerbation of lipid uptake and deposition. Exogenous Ang-(1–7) significantly alleviated renal lipid deposition by downregulating LDLr, SREBP2 and SCAP through its anti-inflammation activity, leading to improvement of proteinuria, blood urea nitrogen, and serum creatinine. Significant renal pathological damage in HFD mice was demonstrated, based on glomerular hypertrophy, serious damage to the glomerular filtration membrane, proliferation of mesangial matrix, infusion of podocytes, tubular cell swelling, tubular dilatation, brush border loss and nuclear loss. Exogenous Ang-(1–7) alleviated this damage through decreasing lipid deposition. Therefore, we propose that Ang-(1–7) exerts renoprotective effects in HFD mice through reducing lipid accumulation by downregulating the LDLr-SREBP2-SCAP pathway.

Renin angiotensin system blockers, ACE inhibitors (ACEI) and angiotensin receptor blockers (ARB) have been broadly used to treat congestive heart failure, hypertension, proteinuria and chronic kidney disease [43–45], and additionally play a significant role in improving dyslipidemia [46,47]. While it is accepted that the ACE2-Ang-(1–7)-Mas axis counteracts the deleterious actions of the ACE-AngII-AT1 axis, the effects of Ang-(1–7) are not limited to its counterregulatory activity. Recent studies have shown a significant effect of Ang-(1–7) in ameliorating lipid metabolic disorders [29,48]. Furthermore, the mechanism underlying Ang-(1–7)-mediated regulation of obesity and dyslipidemia may involve inhibition of the resistin/TLR4/MAPK/NF-ĸB pathway [16]. In addition, in the current study, we observed a significant reduction in body weight and improvement in dyslipidemia induced by a high-fat diet in mice after treatment with Ang-(1–7), indicating that anti-inflammatory effects of Ang-(1–7) may also play a role in ameliorating lipid metabolic disorders.

In summary, we have successfully established a model of high fat-induced lipid metabolic disorders and renal injury. To our knowledge, this study is the first to provide evidence that Ang-(1–7)-mediated regulation of chronic inflammation could ameliorate renal injury induced by lipid metabolism disorders through the LDLr-SREBP2-SCAP pathway. These findings should help to pave the way for developing novel treatments for chronic inflammation and renal diseases in lipid disorders.

## Materials and Methods

### Animals and Diets

Highly specific pathogen-free (SPF) healthy male C57BL/6 mice at 6 weeks of age were obtained from the Animal Center of Chongqing Medical University. Animals were housed in a controlled environment under a 12 h light–12 h dark photoperiod (lights on from 06:00 to 18:00 h) and temperature of 20 ±2°C, with free access to food and water. Mice were randomly divided into four groups (n = 10) and fed the following experimental diets for 12 weeks: standard diet (STD) + saline (STD+saline), HFD (containing 60% kcal fat, D12492 Reseach Diets)+ saline, HFD + Ang-(1–7) and STD + Ang-(1–7),. STD was purchased from the Animal Center of Chongqing Medical University. Ang-(1–7) (144 μg/kg·day) and saline were administered by continuous subcutaneous infusion via osmotic pumps (Model 2002, Alzet) [31] implanted under the dorsal skin of mice for two weeks, followed by sacrifice via decapitation. All efforts were made to minimize animal suffering. The animal study was approved by the Animal Care Committee of Chongqing Medical University, and the experimental procedures complied with the Guidelines for the Care and Use of Laboratory Animals.

### Measurement of Body Weight and food intake

Body weight was measured once a week and food intake was measured twice a week (food intake/body weight) during the treatment period. Mice were sacrificed after overnight fasting, followed by collection of blood and renal tissue samples. Half the renal tissue was immediately frozen on dry ice and stored at −80°C for subsequent analysis, and part of the other half fixed by immersion in 10% formaldehyde for microscopic examination. Small tissue biopsies were punched from the cortex of the remaining slices using a plastic grid with equidistantly spaced holes and fixed with 2.5% glutaraldehyde for electron microscopy examination.

### Serum and Urine analyses

After incubation at room temperature, serum was isolated from blood via centrifugation at 2500 g for 15 min. A proportion of serum was measured for total serum cholesterol (TC), triglycerides (TG), low-density lipoprotein (LDL), C-reactive protein (CRP), blood urea nitrogen (BUN) and serum creatinine (Scr). Urine samples were collected using a metabolic cage for 24 h urinary albumin measurement. All test items of serum and urine were determined using an automatic chemical analyzer (HITACHI 7600, Japan). The remaining serum was used for tumor necrosis factor-alpha (TNF-α) and interleukin-6 (IL-6) measurements. TNF-α was analyzed via enzyme-linked immunosorbent assay (ELISA) and IL-6 detected with chemiluminiscence.

### Oil red O staining

Lipid accumulation in frozen sections of kidney was evaluated with oil red O staining. After fixation in 4% buffered formalin phosphate, dehydration, and embedding in OCT compound (Sakura, USA), renal samples were cut into 7 μm frozen pathological sections. Sections for each group were fixed with 10% formal saline for 30 min, incubated with 1, 2-propanediol for 2 min, stained with Oil Red O for 30 min followed by Carazzi’s hematoxylin for 2 min, and washed in tap water for 5 min. Sections were subsequently examined using light microscopy. Pictures were analyzed using Image Analysis System (Image-Pro Plus v 6.0, Media Cybernetics, Warrendale, PA, USA).

### Measurement of total cholesterol in tissue

Total cholesterol in renal samples from mice was measured using a total cholesterol assay kit (APPLYGEN, Beijing, China) according to the manufacturer’s instructions, at an absorbance of 490 nm on the SpectraMax M2 system (Molecular Devices, USA). The standardized total cholesterol content in each group was relative to that of total tissue proteins determined using the modified Lowry assay.

### HE staining

After fixation in 10% buffered formalin phosphate, dehydration, and embedding in paraffin, renal samples were cut into 3 μm sections for hematoxylin eosin (HE) staining, and observed using an image analysis system (NIS Elements, Nikon, Sendai, Japan). The area of Bowman’s space was measured with the quantitative Image Analysis System (Image-Pro Plus v 6.0, Media Cybernetics, Warrendale, PA, USA). A semi-quantitative assessment was performed to evaluate tubular damage as follows: 0: normal kidney, 1: vacuolation of cytoplasm in <20% tubules, 2: vacuoles in 20–40% tubules, 3: vacuoles in 40–60% of tubules, 4: vacuoles in 60–80% tubules, and 5: >80% tubules with severe vacuolation or tubular atrophy and degeneration [5].

### Transmission Electron Microscopy (TEM)

After fixation with glutaraldehyde, decalcification with EDTA and dehydration in a graded series of ethanol and acetone, the cortex was embedded in epoxy resin and semithin sections generated. A 50 nm section was cut using an ultramicrotome and double-stained with uranyl acetate and alkaline lead citrate, followed by TEM evaluation (Hitachi-7500, Japan).

### Immunohistochemical Reactions

Paraffin tissue sections were cut into 3 μm slices, dewaxed for 10 min twice in xylene, rehydrated through a graded series of ethanol to water, and finally washed three times for 3 min in PBS. Endogenous peroxidase activity was quenched with 3% hydrogen peroxide at room temperature for 10 min, followed by three washes with PBS for 3 min each. After the induced epitope retrieval was heated, sections were washed with another three times with PBS for 3 min, and incubated with Immunol Staining Blocking buffer (P0102. Beyotime) at room temperature for 10 min. Sections were incubated overnight at 4°C with one of the following primary antibodies: anti-LDLr (1:200, Epitomics, USA), anti-SREBP-2 (1:250, Abcam, UK), anti-SCAP (1:100, Abcam, UK), anti-TNF-α (1:250, Abcam, UK), anti-IL-6 (1:400, Abcam, UK) or anti-MCP-1 (1:100, Abcam, UK). Sections were washed with PBS for 5 min three times, followed by incubation with secondary antibody at room temperature for 10 min. After another PBS wash, sections were incubated with 3, 3-diaminobenzidine (DAB) (ZSGB-Bio, China) for 3 min, and counterstained with hematoxylin. Briefly, twenty fields in both glomeruli and tubulointerstitium were randomly used for identification of the positive area under high-power (40×) fields. The percentage of the positive area in the examined field was measured with the quantitative Image Analysis System (Image-Pro Plus v 6.0, Media Cybernetics, Warrendale, PA, USA).

### RNA Extraction and Quantitative RT-PCR

Total RNA from kidney was prepared using a Total RNA Extraction Kit (Bioteke Corporation, China) and used as a template for reverse transcription performed using the RT Reagent Kit (TaKaRa, Japan). GAPDH, LDLr, SCAP, SREBP-2, TNF-α, IL-6, and MCP-1 cDNA were amplified using the specific primers presented in Table 2. Quantitative measurements were performed using SYBR Premix Ex TaqII (TaKaRa, Japan) in an ABI 7300 Real-time PCR System (Applied Biosystems, USA). Real-time quantitative PCR reactions were performed under the following conditions: denaturation at 95°C for 30 sec, 40 cycles of 5 sec at 95°C, and 31 sec at 60°C. The relative comparative CT method was applied to compare gene expression levels between groups using the equation 2^-ΔΔt^. Each sample was run and analyzed at least three times.

**Table 2:** Real-time PCR primer sequences.

Gene	Primer sequences
TNF-α	F GGTGCCTATGTCTCAGCCTCTT
	R GCCATAGAACTGATGAGAGGGAG
IL-6	F TCCAGTTGCCTTCTTGGGAC
	R GTGTAATTAAGCGCCGACTTG
MCP-1	F ACTGAAGCCAGCTCTCTCTTCCTC
	R TTCCTTCTTGGGGTCAGCACAGAC
LDLr	F TTGGGTTGATTCCAAACTCCAT
	R CCGATTGCCCCCATTGA
SREBP2	F CATCCCTTGGGCCAGAAGTT
	R TCCTTGGCTGCTGACTTGATC
SCAP	F AAGGGACCAGGTGGAACACA
	R GCGCGGCCACCTTGTA
GAPDH	F GTCTACTGGTGTCTTCACCACCAT
	R GTTGTCATATTTCTCGTGGTTCAC

### Western blot

Renal tissue was extracted from mice using clean ophthalmic scissors and placed in a homogenizer. Aliquots of protein sample (20 μl) and protein marker (5 μl) were run on SDS-polyacrylamide gels, and transferred onto 0.45 μm polyvinylidene fluoride using Western blot apparatus. Membranes were incubated with 5% BSA (Solarbio, China) in Tris-buffered saline-Tween (TBST) for 1 h at 4°C to block nonspecific binding sites. Immunoblots were incubated with agitation at 4°C overnight in the presence of specific rabbit anti-mouse antibodies against LDLr (1:2000, Epitomics, USA), SREBP-2 (1:500, Abcam, UK), SCAP (1:1000, Abcam, UK), TNF-α (1:2500, Abcam, UK), IL-6 (1:1000, Abcam, UK), MCP-1 (1:2000, Abcam, UK) and GAPDH (1:2000, SAB, USA), followed by three 10 min washes in TBST. After incubation with goat anti-rabbit horseradish peroxidase-conjugated IgG (secondary antibody) for 1 h at room temperature, membranes were subjected to three 10 min washes in TBST. Finally, quantitation of the membranes on autoradiograms was performed using the enhanced chemiluminescence (ECL) kit (KeyGEN Biotech, China) on a Bio-Rad ChemiDoc Imaging System (Bio-Rad, USA).

### Statistical analysis

Values are expressed as means ± SD. Statistical analyses were performed using one-way ANOVA, followed by Bonferroni’s test with SPSS Statistics 17.0 software. Values of *P*<0.05 were considered significant.

## Supporting Information

S1 File The arrive guidelines checklist of animal research. Detailed introduction of each part of this in vivo experiment.
(PDF)
